# When Hysteroscopy Isn’t an Option: Laparoscopic Management of a Symptomatic Submucosal Fibroid

**DOI:** 10.7759/cureus.89270

**Published:** 2025-08-03

**Authors:** Marwa M Elboghdady, Sura Salih, Ahmed M Al Hamoud, Helmi Abdalbari, Wael Hosni

**Affiliations:** 1 Obstetrics and Gynecology, Health and Medical Services (HMS) Al Garhoud Hospital, Dubai, ARE; 2 Medicine, Dubai Medical College, Dubai, ARE; 3 Medicine, Health and Medical Services (HMS) Al Garhoud Hospital, Dubai, ARE

**Keywords:** fibroids, hysteroscopy, laparoscopy, myomectomy, submucosal fibroid

## Abstract

The management of symptomatic uterine fibroids depends on various factors, including patient preferences and individual considerations. The management options are multimodal, including medical and surgical therapies. In this report, we present the case of a 33-year-old female patient with submucosal uterine fibroids managed through laparoscopic myomectomy, chosen over hysteroscopic myomectomy based on the patient's specific preferences and clinical indications. Submucosal fibroids are preferably managed hysteroscopically, while subserosal and large submucosal fibroids are managed laparoscopically. However, to preserve our patient’s virginity and respect her cultural preferences, laparoscopy was used to retrieve the submucosal fibroids. The outcome of the surgery was similar to that of the hysteroscopic approach, with no associated postsurgical complications and a full resolution of the symptoms after follow-up.

## Introduction

Uterine fibroids, also known as uterine leiomyomas, are among the most common benign neoplasms affecting women worldwide. They arise from the smooth muscle lining of the uterus and are subclassified into three types: submucosal, subserosal, and intramural. Although often asymptomatic, uterine leiomyomas can cause a range of symptoms, including heavy menstrual bleeding that leads to anemia and 'bulk symptoms' such as pelvic pain and urinary and bowel dysfunction [1]. The symptoms of uterine fibroids are usually chronic in nature; however, they may very rarely present acutely with severe abdominal pain, necessitating emergency surgery due to torsion of a pedunculated fibroid [2]. Generally, treatment is recommended only for symptomatic fibroids. The specific type of management depends on several factors, including the location, size, and type of fibroids, as well as the patient’s fertility desires. Submucosal uterine fibroids are typically managed via hysteroscopic myomectomy, a minimally invasive approach with favorable patient outcomes [3]. However, cultural preferences may sometimes preclude the use of hysteroscopic methods. In this case report, we present the management of submucosal uterine fibroids via laparoscopic myomectomy instead of the standard hysteroscopic approach, given the size and location of the fibroids. The aim of this case report is to shed light on this alternative approach, which was chosen due to specific patient considerations, and to highlight the importance of individualized treatment plans in gynecological practice.

## Case presentation

A 33-year-old female patient presented with a two-year history of heavy and prolonged menstrual periods, persistent lower abdominal pain, and severe iron deficiency anemia (IDA). The patient had no significant past medical or surgical history apart from a diagnosis of uterine fibroids. The patient denied any history of sexual activity and had no prior gynecological or surgical interventions. She was not taking any regular medications, aside from occasional over-the-counter analgesics and iron supplements. There were no known drug allergies or adverse reactions to medications or anesthesia. On physical examination, the patient appeared in mild discomfort due to lower abdominal pain. Abdominal examination revealed a firm, non-tender mass palpable in the suprapubic region, consistent with an enlarged, irregular uterus.

**Figure 1:**
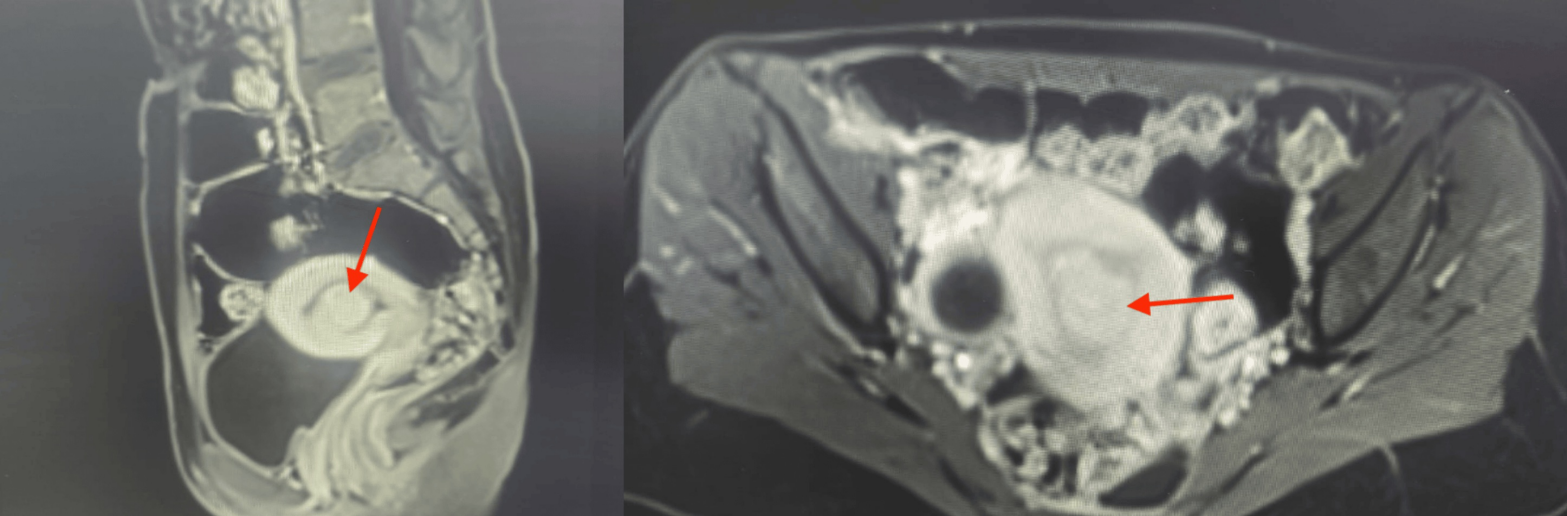
MRI images of the fibroids; the red arrow depicts the submucosal fibroid.

**Figure 2:**
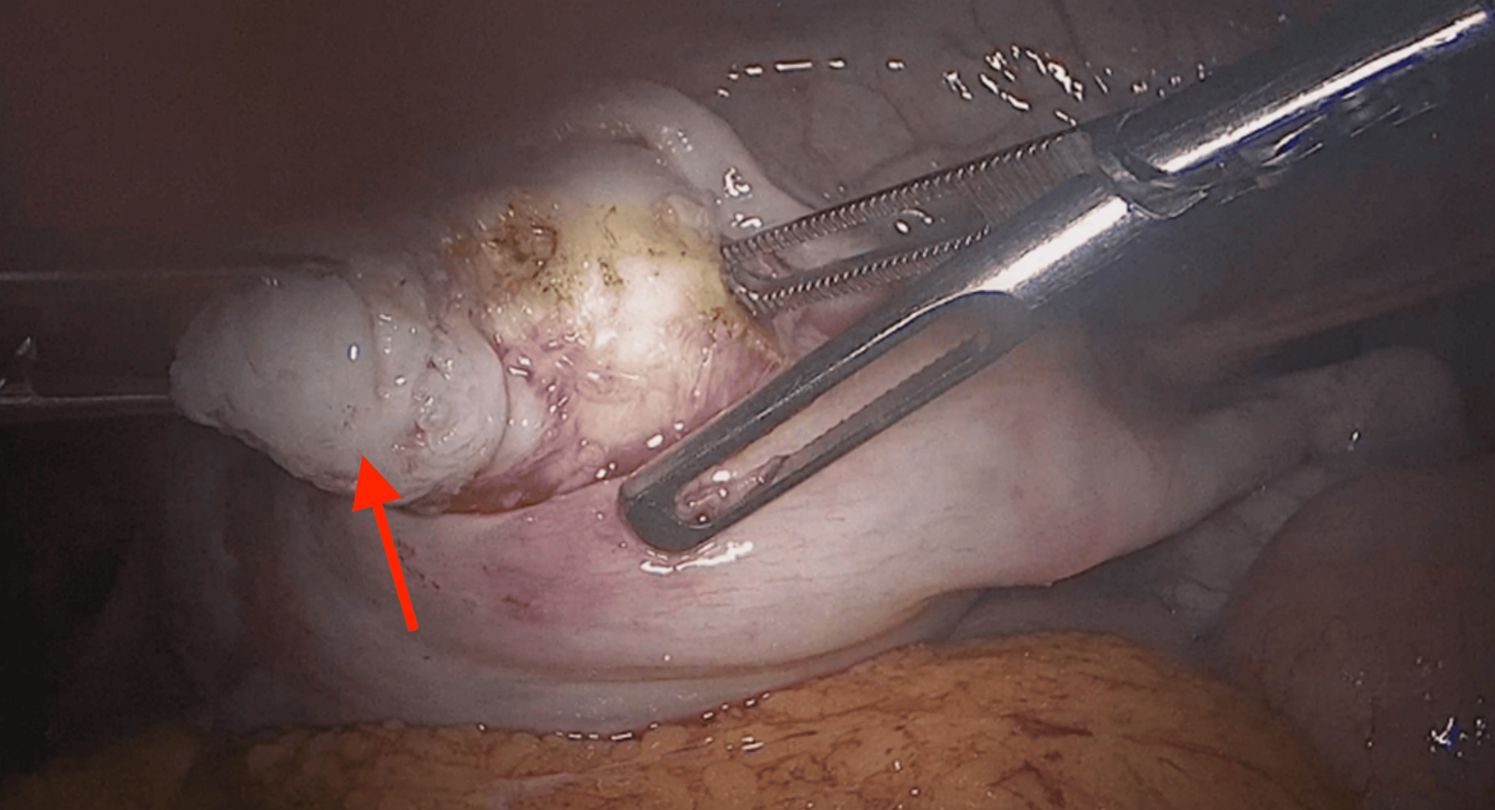
Intraoperative picture of the myomectomy; the red arrow depicts the excised fibroid.

The IDA necessitated multiple administrations of oral and intravenous iron, along with recent blood transfusions at different hospitals. MRI revealed two submucosal fibroids (International Federation of Gynecology and Obstetrics (FIGO) type 0 and type 1, each measuring 2.1 x 2.2 cm), a subserosal fibroid (FIGO type 5, measuring 2.2 x 2 cm), and a large right ovarian cyst (4 x 3 cm) with T2 shading, indicative of an endometriotic hemorrhagic cyst (Figure 1). Apart from the IDA, highlighted by a hemoglobin level of 7 g/dl (normal: 12.5-15), her blood work was normal (Table 1). The surgical management options for the myomectomy were explained to the patient, along with their respective advantages and disadvantages. Due to the patient’s cultural preferences and her virginity, she preferred to avoid the hysteroscopic (vaginal) approach, making the laparoscopic approach the most suitable option for the myomectomy. The surgery was conducted, and the patient did not have any postsurgical complications (Figure 2).

**Table 1:** Blood test results

Test name	Results	Reference range
Hemoglobin	7 g/dL	12.5-15.0 g/dL
Hematocrit	23%	36-46 %
Mean corpuscular volume (MCV)	71 fl	80 - 100 fl
White blood cell count	4.5 x 10^3 /uL	4 - 10 x10^3 /uL
Platelets	239 x 10^3 /uL	150 - 450 x 10^3 /uL
Activated partial thromboplastin time (APTT)	31 seconds	25 - 40 seconds
Prothrombin time (PT)	12.7 seconds	10 - 13.5 seconds
International normalized ratio (INR)	1.09	0.85 - 1.15

## Discussion

The selection among uterine-sparing choices is directed by the size, number, and location of fibroids, the symptoms experienced, and where the patient stands in the reproductive life span. Uterine-preserving interventional treatments include myomectomy, uterine artery embolization, focused ultrasound surgery, and radiofrequency ablation [1]. Laparotomy, laparoscopy, and hysteroscopy are the three modalities utilized to perform a myomectomy. Generally, the abdominal approach, namely laparotomy and laparoscopy, is used to handle subserosal and intramural fibroids. The vaginal approach, through hysteroscopy, is suitable for submucous fibroids [4]. 

Hysteroscopic myomectomy is often the first surgical approach of choice when it comes to submucosal fibroids, especially those that are FIGO type 0, type 1, and some type 2 [3]. The minimally invasive nature of the approach makes it very favorable, with low complication rates and reduced hospitalization periods [3]. This procedure is usually performed on an outpatient basis, typically allowing patients to resume work within a few days and increasing the likelihood of clinical pregnancy. There is insufficient data, however, to demonstrate an association with an increase in the rate of live births [1]. On the other hand, the main complications of hysteroscopic myomectomy include uterine bleeding, perforation, and fluid overload. The risk of surgical re-intervention ranges between 10% and 35%, which may involve a repeat hysteroscopic myomectomy, open myomectomy, or even hysterectomy [5]. 

With respect to surgical approaches, myomectomy, whether via laparotomy or laparoscopy, is a viable option to address one or multiple fibroids, alleviate bulk and bleeding symptoms, and preserve fertility [1]. There are certain circumstances where laparoscopy is preferred for the removal of submucosal fibroids. If the tissue separating the fibroid from the uterine serosa is too thin, a myoma grasper can potentially result in uterine perforation. In such cases, intervention via laparotomy or laparoscopy is necessary to repair the uterine defect. Therefore, it is not advised to perform a hysteroscopic myomectomy when the intramural part of the fibroid is greater than 4 cm in diameter and the thickness of the myometrium at the implantation site is less than 5 mm [4]. In addition, if the intramural extent of the myoma is greater than 50% or if there are concomitant non-submucous fibroids, laparoscopic myomectomy is recommended. The concerns regarding laparoscopic myomectomy of submucous fibroids primarily include breaching the integrity of the endometrial cavity, healing of the suture line, and the likelihood of later pregnancy with the associated risk of uterine rupture. The principal causes of dehiscence may include suboptimal suturing of the uterine incision, poor wound healing due to excessive use of coagulation or any tissue-damaging tools. Studies indicate that if the myometrium is effectively sutured without tension, the risk of uterine rupture does not increase significantly even if the endometrial cavity is opened during surgery. It is best to avoid the unnecessary use of thermal energy for hemostasis due to its potential to impair vascularization and cause tissue necrosis, thereby increasing the risks of uterine rupture and fistula formation [5]. The location of submucous fibroids within the uterus significantly influences whether hysteroscopic removal is feasible. A large sessile fundal fibroid, for instance, may be inaccessible to the resectoscope due to its design limitations. In such cases, partial fibroid resection may be attempted initially, but subsequent surgeries could be necessary. Therefore, transabdominal myomectomy is the definitive modality in this case. For combined removal of intramural and subserosal fibroids, resection solely by hysteroscopy is impossible and will leave an anatomically distorted uterus. Utilizing laparoscopy for treating sizable submucous fibroids can avoid the challenges and enable comprehensive fibroid removal in a single procedure [4].

A retrospective analysis study compared the outcomes of hysteroscopic and laparoscopic myomectomy in 85 patients with type II submucous myomas. Both procedures were found to be effective in treating the condition, but hysteroscopic myomectomy offered the advantages of shorter postoperative recovery times and hospital stays. There were no significant differences in operation time, intraoperative bleeding, or complication rates between the two methods. Therefore, while both surgical approaches are viable, hysteroscopic myomectomy is preferable when feasible due to its quicker recovery and reduced hospital stay. Laparoscopic operation has higher advantages in type II submucous myomas of greater than 4 cm in diameter, whereas hysteroscopic operation has higher advantages in type II submucous myomas of less than 4 cm in diameter [6].

Moreover, the postoperative outcomes for laparoscopic myomectomy of submucosal fibroids are like those of other types of fibroids. In a retrospective cohort study that evaluated 350 patients who underwent laparoscopic myomectomy for symptomatic uterine fibroids, there were no reported significant differences between the outcomes for the 33 patients with submucous fibroids and the 317 patients with other fibroids in different locations in terms of perioperative outcomes and immediate complications between the groups [7].

Typically, hysteroscopy is the method of choice for management of submucosal fibroids, as it directly accesses the fibroids within the uterine cavity. However, our patient, an unmarried female with no history of sexual activity, has cultural considerations that make the use of a hysteroscope unacceptable. However, given this cultural restriction, laparoscopic myomectomy emerges as a viable substitute for the small submucosal fibroids, albeit it requires surgical expertise. It is of note that the size of these fibroids is smaller than the threshold typically recommended for laparoscopic intervention. Uterine artery embolization is a possible alternative, but concerns regarding potential ovarian dysfunction and future pregnancies limit its practicability in our case. Thus, careful consideration of cultural, anatomical, and clinical factors is essential in determining the most appropriate management strategy.

## Conclusions

The management of uterine fibroids depends on a multifactorial approach that is tailored to fit the patient’s best interests while taking into account the anatomical characteristics, surgical feasibility, and cultural sensitivities. Hysteroscopic myomectomy remains the gold standard for treating accessible submucosal fibroids due to its minimally invasive nature and favorable recovery profile. However, it is not universally applicable. In circumstances where hysteroscopy is contraindicated or infeasible, as in the presented case, laparoscopic myomectomy, though relatively challenging, offers a viable and effective alternative, provided that surgical expertise is available.
